# Salivary cortisol dynamics and their relationship with sleep and mental well-being in adults receiving a phospholipid-based *Melissa officinalis* supplement: a secondary analysis in a subpopulation

**DOI:** 10.29219/fnr.v70.14023

**Published:** 2026-05-13

**Authors:** Giuseppe Mazzola, Mariangela Rondanelli, Alessandro Lazzarotti, Paola Misiano, Giovanna Petrangolini, Simone Perna

**Affiliations:** 1 Endocrinology and Nutrition Unit, Azienda di Servizi alla Persona “Istituto Santa Margherita”, University of Pavia, Pavia, Italy; 2 Department of Public Health, Experimental and Forensic Medicine, University of Pavia, Pavia, Italy; 3 Department of Pharmacological and Biomolecular Sciences, Università Degli Studi di Milano, Milan, Italy; 4 Medical Department, Indena S.p.A., Milan, Italy; 5 Division of Human Nutrition, Department of Food, Environmental and Nutritional Sciences (DeFENS), University of Milan, Milan, Italy

**Keywords:** Melissa, lemon balm, cortisol modulation, mental well-being, Relissa, Phytosome, phospholipids, HPA axis, rosmarinic acid

## Abstract

**Background:**

Dysregulation of the hypothalamic–pituitary–adrenal (HPA) axis, as indicated by altered salivary cortisol secretion, has been linked to poor sleep quality, mood disturbances, and emotional distress. In the principal clinical trial, 3 weeks of Melissa phospholipids’ supplementation was associated with significant improvements in sleep quality and mood outcomes.

**Objective:**

This study aimed to evaluate salivary cortisol dynamics in a subpopulation supplemented with *Melissa officinalis* extract formulated in phospholipids for 3 weeks.

**Design:**

This secondary analysis evaluated a predefined subgroup (*n* = 12) receiving 400 mg/day of Melissa phospholipids for 3 weeks. Salivary cortisol was collected at 3 timepoints at baseline (T0), week 1 (T1), and week 3 (T2), and quantified by high-sensitivity enzyme-linked immunosorbent assay (ELISA). Associations between cortisol levels and psychometric outcomes were also assessed.

**Results:**

Mean salivary cortisol levels decreased progressively over the 3-week supplementation period, with a significant main effect of time (*P* < 0.001). Significant correlations (*P* < 0.05) were also observed between psychometric domains, including PSQI, DASS-21 depression and stress, PANAS, WEMWBS, and WHOQoL-BREF scores, indicating consistent improvements in sleep quality, mood, and perceived well-being.

**Discussion and conclusions:**

This secondary analysis provides preliminary evidence that Melissa phospholipids supplementation, is associated with a significant modulation of HPA axis activity, characterized by a reduction in daily salivary cortisol levels, in adults with poor sleep quality and emotional distress.

## Popular scientific summary

- High cortisol levels were associated with low sleep, mood disturbances, and emotional distress.
- Melissa phospholipids supplementation modulated daily salivary cortisol levels in adults with poor sleep quality and emotional distress.
- Melissa phospholipids supplementation was associated with a reduction in daily salivary cortisol levels, indicating a favorable modulation of HPA axis activity.
- The modulation of HPA axis activity achieved through the reduction of salivary cortisol levels, was correlated with improvements in sleep quality and mental well-being scores due to Melissa phospholipids supplementation.

Dysregulation of the hypothalamic–pituitary–adrenal (HPA) axis, as reflected by altered diurnal cortisol secretion, is strongly implicated in the pathophysiology of sleep disturbances, mood disorders, and stress-related conditions (1–5). Elevated or blunted cortisol levels have been associated with impaired sleep quality, heightened emotional distress, and reduced mental well-being in both clinical populations and non-clinical models (1, 2, 6). Given this bidirectional relationship, interventions capable of modulating HPA axis activity may have a significant impact on sleep and stress resilience.

*Melissa officinalis* L. (lemon balm) is a botanical extract widely utilized for its sleep and calming promoting effects. Standardized extracts of *M. officinalis*, particularly when formulated with phospholipid-based delivery systems, have demonstrated beneficial effects on sleep quality, perceived stress, and mood in adults experiencing emotional distress (7–10). These effects are thought to be mediated by multiple mechanisms, including GABAergic modulation, anti-inflammatory and antioxidant properties, and regulation of the HPA axis (11, 12). Experimental models suggest that rosmarinic acid, the main bioactive compound of *M. officinalis*, can reduce stress-induced corticosterone elevations, which may translate into improved sleep architecture and psychological outcomes (8, 13).

In a recent clinical study, 3 weeks of daily supplementation with Melissa extract formulated in phospholipids (Melissa Phytosome™, as Relissa™) was associated with significant improvements in sleep quality, depression, stress, affective states, and quality of life in adults with emotional distress at the daily dose of 400 mg (14–16). Importantly, these findings demonstrated a dose–response relationship and a rapid onset of action for the daily 400 mg dose, underscoring the potential of this formulation as a short-term efficient support. However, whether these benefits are paralleled by changes in cortisol secretion remains unclear. Salivary cortisol provides a non-invasive, reliable measure of HPA axis function and can capture both absolute levels and dynamic changes over time (16, 17).

The association between HPA axis modulation and improvements in sleep and psychological/mental well-being has been hypothesized but rarely demonstrated in a nutraceutical intervention context. However, a very recent review supported nutritional supplements as potentially useful in restoring HPA axis balance (3). A better understanding of this link could clarify the biological mechanisms underlying the clinical benefits of *M. officinalis* and update the design of targeted supplements for healthy individuals or with stress-related sleep disturbances. Moreover, exploring correlations between cortisol and validated psychometric measures may help to differentiate effects mediated by sleep restoration from those driven by direct neuroendocrine actions.

This secondary analysis of our principal study (14) aimed to investigate the effects of the supplementation of *M. officinalis* extract formulated in phospholipids (MOP) on salivary cortisol dynamics and to explore their relationship with validated measures of sleep quality (PSQI) and psychological well-being (DASS-21, PANAS, WEMWBS, WHO-QoL-BREF). We hypothesized that this supplementation would result in modulation of salivary cortisol levels across the 3-week intervention and that changes in cortisol would correlate with improvements in sleep quality and mental well-being outcomes.

## Materials and methods

### Study design and participants

This was a prospective secondary analysis conducted on a predefined subgroup of 12 adult participants enrolled in the primary clinical study previously reported (ClinicalTrial.gov ID: NCT06942897) (14). Participants were those who consented to additional sampling and subgroup formation was consent-based. The study was conducted according to the guidelines of the Declaration of Helsinki and approved by the Institutional Ethics Committee of University of Pavia (Ethical code number: 1267/21092023). ClinicalTrial.gov code NCT06942897. Informed consent was obtained from all subjects involved in the study. All participants in this subgroup received daily supplementation with 400 mg/day of Melissa extract formulated in phospholipids (hereafter Melissa phospholipids, as Melissa Phytosome™ or Relissa™, Indena S.p.A, Italy) for 3 consecutive weeks. The 400 mg daily dose and the 3-week supplementation of Melissa Phospholipids were selected based on previous clinical evidence (9, 10, 15) demonstrating that *M. officinalis*, particularly as phospholipids-based formulation, exerts significant effects on sleep quality and emotional distress at that daily dose within this timeframe. Furthermore, recent findings suggested a rapid onset of action for the 400 mg dose, justifying a short-term evaluation of its neuroendocrine impact (14). Eligibility criteria and study procedures were identical to those described in the primary trial, and inclusion criteria comprised an age range of 18–70 years, poor sleep quality defined by a Pittsburgh Sleep Quality Index (PSQI) score >5, and/or clinically relevant emotional distress as indicated by elevated Depression, Anxiety, and Stress Scale (DASS-21) scores (depression ≥14, anxiety ≥10, stress ≥19). Exclusion criteria included major psychiatric disorders, untreated endocrine diseases, severe chronic medical conditions, and current use of medications or supplements known to affect sleep or cortisol secretion, as reported in Table 1.

**Table 1:** Inclusion and exclusion criteria

Inclusion criteria	Exclusion criteria
Adults aged 18–70 years	Major psychiatric disorders
Poor sleep quality (PSQI score > 5)	Untreated endocrine diseases
Clinically relevant emotional distress (DASS-21: depression ≥14, anxiety ≥10, stress ≥19)	Severe chronic medical conditions
Ability to provide informed consent	Current use of medications or supplements affecting sleep or cortisol secretion

### Supplementation

Participants received two tablets per day of Melissa Phospholipids (200 mg x 2) (MOP as Melissa Phytosome™, Relissa™, donated by Indena S.p.A, Italy). administered orally in the evening, approximately 30 to 60 min before bedtime, with a glass of water. Each lactose- and gluten-free tablet contained 200 mg of MOP. MOP is a *M. officinalis* L. leaf aqueous extract formulated as a solid dispersion into a sunflower lecithin-based food-grade matrix according to Phytosome™ technology. The solid dispersion of sunflower lecithin with Melissa extract prevents the self-aggregation of the botanical and eliminates a primary barrier to the absorption of the phytonutrients. MOP is standardized to contain 17%–23% hydroxycinnamic acid derivatives and analyzed for its rosmarinic acid content.

Compliance was monitored by tablets count at each visit, and all participants included in the analysis demonstrated a compliance rate > 90%. No changes in diet, lifestyle, or concomitant medications were permitted during the study period.

### Salivary cortisol assessment

Salivary cortisol was evaluated as an exploratory biomarker to investigate potential modulation of HPA axis activity associated with MOP supplementation. Sampling was performed in a subset of 12 participants from the 400 mg/day supplementation group who consented to additional testing. Unstimulated saliva samples were collected using standardized collection devices (Salivette^®^, Sarstedt AG & Co., Nümbrecht, Germany) at three specific time points, 08:00 am, 12:00 pm, and 6:00 pm, to capture the diurnal cortisol profile. Collections were conducted at baseline (T0), after 1 week (T1), and after 3 weeks of supplementation (T2). Participants were instructed to refrain from eating, drinking (except water), brushing their teeth, or smoking for at least 30 min before each sampling.

Samples were immediately stored at −20°C until analysis. Cortisol concentrations were determined in duplicate using a high-sensitivity enzyme-linked immunosorbent assay (ELISA; Salimetrics^®^, State College, PA, USA), with a lower limit of detection of 0.007 µg/dL and intra- and inter-assay coefficients of variation below 8%. Reference values for morning salivary cortisol in healthy adults were considered to range between 0.15 and 0.60 µg/dL (16).

### Psychometric assessments

To explore the relationship between HPA axis activity and clinical outcomes, participants also completed the same battery of validated psychometric instruments used in the primary study at T0, T1, and T2. These included the PSQI for sleep quality, DASS-21 for depression, anxiety, and stress, the Positive and Negative Affect Schedule (PANAS), the Warwick-Edinburgh Mental Well-Being Scale (WEMWBS), and the World Health Organization Quality of Life-BREF (WHO-QoL-BREF) (18–21).

### Outcomes

The primary outcome of this secondary analysis was the change in salivary cortisol concentrations over the 3-week supplementation period. Secondary outcomes included the correlations between changes in cortisol levels and changes in psychometric scores for sleep quality and mental well-being.

### Statistical analysis

Descriptive statistics were expressed as mean ± standard deviation (SD) or median (interquartile range) for continuous variables, and as frequencies and percentages for categorical variables. Normality of distributions was assessed using the Shapiro–Wilk test. For the evaluation of the overall daily adrenal output, an aggregated daily mean cortisol value was derived for each participant at each time point (T0, T1, T2). This value was calculated as the mean of the samples collected during the day (08:00, 12:00, and 18:00). This aggregated measure was used for subsequent longitudinal analyses and correlations with psychometric scores. For salivary cortisol, the effect of time across the three assessments (T0, T1, T2) was evaluated using a one-way repeated-measures ANOVA (ANalysis Of VAriance), followed by Sidak’s post hoc tests for pairwise comparisons (T0 vs. T1, T0 vs. T2, T1 vs. T2). Where appropriate, sphericity was assessed (Mauchly’s test) and Greenhouse–Geisser correction was applied. For mean differences, the 95% confidence interval (95% CI) was also calculated. Within the salivary-cortisol subset, changes in psychometric scores over time (primarily T0 vs. T2) were analyzed using the paired t-test or the Wilcoxon signed-rank test, as appropriate based on normality. All tests were two-tailed, and a *P*-value < 0.05 was considered statistically significant. To account for the risk of Type I error inflation due to the high number of pairwise correlations (*n* = 276), the Benjamini-Hochberg False Discovery Rate (FDR) procedure was applied. Correlation results were categorized as ‘Significant’ (*P*_adj_ < 0.05) or ‘Exploratory’ (uncorrected *P* < 0.05). Additionally, correlation analyses were performed on change scores (ΔT2–T0) to explore the mechanistic link between cortisol reduction and psychometric improvements.

Statistical analyses were performed using GraphPad Prism version 10.0 (GraphPad Software, San Diego, CA, USA).

## Results

### Baseline characteristics of the subgroup

The subgroup included in this secondary analysis consisted of 12 participants (6 females and 6 males) who daily received 400 mg (2 × 200 mg/tablet) Melissa phospholipids and underwent salivary cortisol assessment at baseline (T0), week 1 (T1), and week 3 (T2). The mean age of participants was 52.08 ± 6.64 years, and the mean BMI was 25.70 ± 2.86 kg/m^2^. Baseline demographic and clinical characteristics are summarized in Table 2.

**Table 2:** Baseline demographic, clinical, and psychometric characteristics of the subgroup at baseline

Subjects’ characteristic (*n* = 12)	Value
Age (years)	52.08 ± 6.64
Sex (M/F)	6 / 6
BMI (kg/m^2^)	25.70 ± 2.86
PSQI total score	8.17 ± 1.40
DASS-21 Depression	21.58 ± 3.00
DASS-21 Anxiety	20.17 ± 1.40
DASS-21 Stress	20.83 ± 1.75
PANAS-Positive	13.92 ± 1.78
PANAS-Negative	15.00 ± 0.95
WEMWBS total score	37.67 ± 3.08
WHO-QoL-BREF total score	4.83 ± 1.19
Morning salivary cortisol (µg/dL)	0.2430 ± 0.0231

Values are expressed as mean ± standard deviation (SD) or number (%). Comparisons between groups were performed using unpaired t-tests for continuous variables and chi-squared tests for categorical variables.

At baseline, mean PSQI total score was 8.17 ± 1.40, indicating poor sleep quality in all participants. Mean DASS-21 depression, anxiety, and stress scores were 21.58 ± 3.00, 20.17 ± 1.40, and 20.83 ± 1.75, respectively, consistent with mild-to-moderate emotional distress. Baseline morning salivary cortisol levels were 0.2430 ± 0.0231 µg/dL, which is in the reference range (0.15–0.60 µg/dL).

No participants discontinued the intervention, and adherence to the supplementation protocol was excellent, with a mean compliance rate of 95%.

### Changes in salivary cortisol levels over time

The changes in salivary cortisol concentrations are summarized in Table 3. These were measured at different time points (08:00 am, 12:00 pm, and 6:00 pm) and as aggregated daily means during the 3-week supplementation period with Melissa phospholipids at 400 mg/day. Statistical analyses were performed on 12 valid cases using one-way repeated-measures ANOVA with Sidak’s post hoc corrections.

**Table 3:** Changes in salivary cortisol levels (hourly and aggregate means) following supplementation with Melissa phospholipids (400 mg)

Variable/time point	Mean ± SD	RM-ANOVA F (df) / η*p*2	
Aggregated mean cortisol levels	mg/dL	*F*(2,33) = 9.56∗∗∗ / 0.465	
Baseline (T0)	0.2430 ± 0.0231	*P* < 0.001	
Week 1 (T1)	0.2335 ± 0.0222		
Week 3 (T2)	0.2043 ± 0.0225		
Cortisol levels at 8:00 am		*F*(2,22) = 9.696∗∗ / 0.469	
Baseline (T0)	0.2381 ± 0.02883		
Week 1 (T1)	0.2279 ± 0.02630		
Week 3 (T2)	0.2004 ± 0.04063		
Cortisol levels at 12:00 pm		*F*(2,22) = 45.399∗∗∗ / 0.805	
Baseline (T0)	0.2414 ± 0.02266		
Week 1 (T1)	0.2397 ± 0.02446		
Week 3 (T2)	0.2107 ± 0.02117		
Cortisol levels at 6:00 pm		*F*(2,22) = 78.381∗∗∗ / 0.877	
Baseline (T0)	0.2496 ± 0.02222		
Week 1 (T1)	0.2328 ± 0.02202		
Week 3 (T2)	0.2017 ± 0.02430		
*Post hoc* comparisons (Sidak’s test) *P*-values	T0 vs. T1	T0 vs. T2	T1 vs. T2
Aggregated Cortisol Data	*P* > 0.05	*P* < 0.001∗∗∗	*P* < 0.05∗
Cortisol 8:00 am	*P* < 0.01∗∗	*P* < 0.05∗	*P* > 0.05
Cortisol 12:00 pm	*P* > 0.05	*P* < 0.001∗∗∗	*P* < 0.001∗∗∗
Cortisol 6:00 pm	*P* < 0.005∗∗	*P* < 0.001∗∗∗	*P* < 0.001∗∗∗

Values are expressed as mean ± SD. Statistical analysis was performed using one-way repeated-measures ANOVA and Sidak’s *post hoc* tests. *N*: Number of valid cases (listwise) = 12. *F*: *F*-value of the RM-ANOVA test. ηp2: Partial Eta Squared (Effect Size). Statistical significance of the *F* tests and *post hoc* comparisons.

Aggregated-Daily Mean Cortisol: The overall mean salivary cortisol levels decreased progressively across the 3 weeks of supplementation, showing a significant main effect of time (*F*(2,33) = 9.56, 95% CI: −0.016 to 0.004, *P* < 0.001). Mean cortisol levels declined from 0.2430 ± 0.0231 at baseline to 0.2335 ± 0.0222 after 1 week and to 0.2043 ± 0.0225 at week 3. Post hoc comparisons revealed a significant reduction between week 0 and week 3 (95% CI: −0.016 to 0.004, *P* < 0.001) and between week 1 and week 3 (95% CI: −0.0006 to 0.011, *P* < 0.05). While the aggregated daily mean changes from week 0 to week 1 did not reach statistical significance (95% CI: −0.021 to 0.004, *P* > 0.05), a significant reduction was already detectable at the 08:00 am time-point after only 1 week of supplementation (95% CI: −0.015 to 0.0009, *P* < 0.01), as shown in Table 3.

**Fig. 1:**
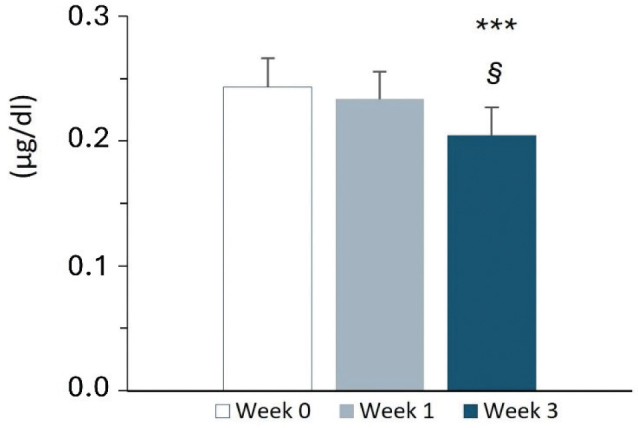
Salivary cortisol levels measured 3 times / day, at basal (T0, pre supplementation, white bar), after one week (T1, gray bar) and 3 weeks (T2, blue bar) of 400 mg Melissa phospholipids supplementation. Each bar is the mean ± SD of *n* = 12 subjects. ***: *P* < 0.001 versus T0, §*P* < 0.05 versus T1, respectively (Sidak’s test).

Collectively, salivary cortisol analyses indicate a progressive and statistically robust reduction in cortisol secretion over the 3-week supplementation period (Fig. 1). These findings are consistent with a downregulation of HPA axis activity within the physiological range and align with the concurrent improvements observed in sleep quality, mood regulation, and psychological well-being, suggesting that changes observed during MOP supplementation are associated with both behavioral and neuroendocrine mechanisms.

**Fig. 2:**
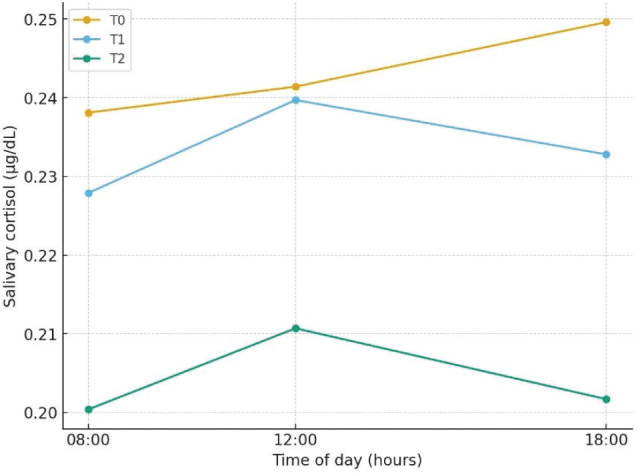
Changes in Salivary Cortisol levels (Hourly). Salivary cortisol levels (mean) at baseline (T0 in yellow), after 1 week (T1 in blue) and after 3 weeks (T2 in green) of supplementation.

To further characterize the diurnal pattern of cortisol secretion, pairwise comparisons were performed between sampling times (08:00, 12:00, and 18:00) within each assessment week (Table 3 and Fig. 2). At baseline, cortisol levels were significantly higher at 08:00 compared with both 12:00 (95% CI: 0.004–0.016, *P* = 0.008) and 18:00 (95% CI: 0.013–0.061, *P* = 0.017), while the difference between 12:00 and 18:00 did not reach significance (*P* = 0.066). After 1 week, cortisol values remained comparable between 08:00 and 12:00 (95% CI: 0.004–0.050, *P* = 0.926), but decreased significantly at 18:00 compared with both earlier time points (*P* < 0.001). By week 3, while a significant overall reduction in cortisol was observed, the diurnal profile remained relatively flat, with significantly higher levels at 08:00 than at 12:00 (95% CI: 0.008–0.025, *P* = 0.003) and 18:00 (95% CI: 0.038–0.057, *P* < 0.001), and higher values at 12:00 than at 18:00 (95% CI: 0.023–0.038, *P* < 0.001). These results suggest a progressive reduction in the overall daily cortisol burden (area under the curve/aggregated mean) (16).

### Correlations between changes in salivary cortisol and psychometric outcomes

To examine potential associations between neuroendocrine modulation and mental outcomes, paired-sample analyses were conducted between morning salivary cortisol levels and psychometric measures assessing sleep quality, mood, and well-being (PSQI, DASS-21, PANAS, WEMWBS, and WHOQoL-BREF). Data are reported in Tables 4 and 5.

**Table 4:** Correlations between psychometric outcomes (paired samples, *P* < 0.05)

Psychometric Pair	Correlation (*r*)	*P* (uncorr.)	Adj. *P*-value (FDR)
Intra-scale associations (Stability)			
DASS-21 Depression T2 & T0	0.923	< 0.001	< 0.001*
WHOQoL Physical Health T2 & T0	0.909	< 0.001	< 0.001*
WEMWBS T2 & T0	0.830	0.001	0.023*
WHOQoL T2 & T0	0.825	0.001	0.023*
PANAS Positive T2 & T0	0.782	0.003	0.054 (Trend)
PANAS Negative T2 & T0	0.690	0.013	0.200
PSQI T2 & T0	0.633	0.027	0.359
Cross-scale associations (Exploratory)			
PSQI T0 & WHOQoL Physical Health T0	0.716	0.009	0.136
DASS-21 Stress T0 & PANAS PosT2	0.683	0.014	0.187
PSQI T2 & WHOQoL Environment T0	-0.687	0.014	0.187
DASS-21 Depression T2 & PANAS Neg T2	0.650	0.022	0.291
WEMWBS T2 & WHOQoL Physical Health T0	0.622	0.031	0.359

Positive correlation (*r* > 0) indicates direct association; negative correlation (*r* < 0) indicates inverse relationship.

**Table 5:** A summary of significant relationships (*P* < 0.05)

Outcome / relationship	Direction	Strength (*r*)	*P* (uncorr.)	Status (FDR)
Intra-scale associations (stability)				
DASS-21 Depression (T2 vs. T0)	Positive	0.923	< 0.001	Significant
WHOQoL Physical Health (T2 vs. T0)	Positive	0.909	< 0.001	Significant
WEMWBS Well-being (T2 vs. T0)	Positive	0.830	0.001	Significant
WHOQoL Total Score (T2 vs. T0)	Positive	0.825	0.001	Significant
PANAS Positive (T2 vs. T0)	Positive	0.782	0.003	Trend
PSQI Sleep Quality (T2 vs. T0)	Positive	0.633	0.027	Exploratory
Psychophysiological links (cross-scale)				
PSQI ↔ WHOQoL Physical Health	Positive	0.716	0.009	Exploratory
DASS-21 Stress ↔ PANAS Pos	Positive	0.683	0.014	Exploratory
PSQI ↔ WHOQoL Environment	Negative	-0.687	0.014	Exploratory
DASS-21 Depression ↔ PANAS Neg	Positive	0.650	0.022	Exploratory
Mechanistic trends (delta scores)				
Δ Cortisol ↔ Δ Stress (T2-T0)	Positive	0.354	0.259	Exploratory
Δ Cortisol ↔ Δ Sleep (T2-T0)	Positive	0.298	0.347	Exploratory

T0 = baseline; T2 = week 3; Δ= change between T0 and T2. ‘Significant’ indicates results surviving Benjamini-Hochberg False Discovery Rate (FDR) correction. ‘Exploratory’ indicates directional associations with uncorrected *P* < 0.05 or mechanistic trends that did not reach adjusted significance due to the limited sample size (*n* = 12).

Several statistically significant correlations (*P* < 0.05) were identified across these domains (Table 5). Within the sleep domain, the PSQI total score at week 3 showed a strong positive association with baseline PSQI values (*r* = 0.633, *P* = 0.027), indicating consistent individual patterns of sleep quality over time. Conversely, PSQI at week 3 was inversely correlated with both PANAS Negative Affect at baseline (*r* = −0.585, *P* = 0.046) and the WHOQoL environmental domain at week 3 (*r* = −0.687, *P* = 0.014), suggesting that better sleep quality was related to lower negative affect and improved perceived environmental well-being. Baseline PSQI scores were also positively correlated with WHOQoL physical health, both at week 3 (*r* = 0.668, *P* = 0.018) and baseline (*r* = 0.716, *P* = 0.009).

Within the affective and stress-related measures, high intra-scale correlations were observed for DASS-21 subscales, confirming strong temporal consistency across time points. Specifically, depression (*r* = 0.923, *P* < 0.001), anxiety (*r* = 0.677, *P* = 0.016), and stress scores displayed significant internal stability. Similar patterns were observed for PANAS Positive (*r* = 0.782, *P* = 0.003) and PANAS Negative (*r* = 0.690, *P* = 0.013), as well as for overall psychological well-being measured by WEMWBS (*r* = 0.830, *P* = 0.001) and general quality of life assessed by WHOQoL-BREF (*r* = 0.825, *P* = 0.001).

Beyond intra-scale relationships, several relevant cross-scale associations emerged. DASS-21 Depression at week 3 correlated positively with PANAS Negative Affect at the same time point (*r* = 0.650, *P* = 0.022) and negatively with WHOQoL Interpersonal Relationships (*r* = −0.596, *P* = 0.041). DASS-21 Depression at baseline was also positively associated with PANAS Negative Affect at week 3 (*r* = 0.617, *P* = 0.033). Similarly, DASS-21 Stress at baseline correlated positively with PANAS Positive Affect at week 3 (*r* = 0.683, *P* = 0.014) but inversely with WHOQoL Environment (*r* = −0.585, *P* = 0.046).

Other noteworthy associations were found between PANAS Positive Affect at baseline and the WHOQoL environmental domain at week 3 (*r* = 0.587, *P* = 0.045), as well as between PANAS Negative Affect and environmental well-being at week 3 (*r* = 0.594, *P* = 0.042). Measures of global well-being were also interrelated, with WEMWBS at week 3 correlating positively with WHOQoL Physical Health (*r* = 0.622, *P* = 0.031). Within the WHOQoL subdomains, strong associations were observed between Physical Health at week 3 and baseline values (*r* = 0.909, *P* < 0.001), and between Mental Health at week 3 and baseline (*r* = 0.681, *P* = 0.015).

Paired *t*-tests confirmed significant within-subject improvements (*P* ≤ 0.007) for the main psychometric scales between baseline and week 3, including PSQI, DASS-21 Depression, WEMWBS, and WHOQoL- BREF. Nearly all cross-scale comparisons were statistically significant (*P* < 0.05), with only about 20 out of 276 pairs not meeting the significance threshold. Non-parametric Wilcoxon signed-rank tests yielded consistent results, identifying four significant differences between week 3 and 1 (*Z* ranging from −2.958 to −3.076, all *P* ≤ 0.003), thereby confirming the robustness of the observed changes across measures.

Strong positive correlations were confirmed after FDR correction for intra-scale stability, such as for salivary cortisol (*r* = 0.949, *P*_adj_ < 0.001) and BMI. Regarding cross-domain associations (e.g. cortisol vs. DASS-21 scores), several correlations were observed (*P* < 0.05), but did not reach significance after FDR adjustment.

## Discussion

This secondary exploratory analysis of the principal study (14) provides further insight into the neuroendocrine mechanisms potentially associated with the observations recorded during Melissa phospholipids supplementation. The results show that 3 weeks of daily administration of 400 mg Melissa phospholipids were associated with a progressive and significant reduction in salivary cortisol levels, in adults presenting with poor sleep quality and mild-to-moderate emotional distress. These findings suggest that the improvement in sleep quality and mental well-being observed in the primary trial may be, at least in part, a favorable modulation of HPA axis activity. Salivary cortisol concentrations declined gradually across the 3-week period, with mean aggregated levels decreasing by approximately 16% from baseline. By the end of supplementation, three-quarters of participants exhibited cortisol values within the physiological reference range, indicating a progressive shift in HPA axis activity toward lower physiological levels. This trajectory mirrors patterns reported for other nutraceutical or behavioral interventions targeting stress and sleep dysregulation, such as ashwagandha extract, L-theanine, or mindfulness-based stress reduction (8, 16). Participants exhibited a flattened diurnal cortisol pattern at baseline, followed by a gradual reappearance of morning–evening variability after treatment. Thus, the within-day comparisons confirmed this trend, showing a gradual reappearance of a physiological diurnal pattern. Whereas baseline data reflected a blunted cortisol rhythm–typical of chronic stress or HPA axis dysregulation–subsequent measurements revealed a stepwise restoration of morning–evening variability. By week 3, the overall daily cortisol output was significantly lower, although the typical morning-to-evening decline was not fully re-established. This suggests that MOP supplementation primarily acts by lowering the total cortisol burden rather than specifically remodeling the circadian architecture in this timeframe.

Correlation analyses provided additional insight into the psychophysiological interplay between endocrine modulation and subjective well-being. Within the sleep domain, PSQI total score at week 3 showed a significant positive association with baseline PSQI, confirming consistent individual sleep profiles, but was inversely correlated with both PANAS Negative Affect and the WHOQoL environmental domain. This pattern indicates that participants who reported greater improvements in sleep quality also tended to experience reduced negative affect and better perceived environmental comfort. Within the affective dimension, DASS-21 Depression demonstrated robust temporal stability and significant cross-scale correlations with PANAS Negative Affect and WHOQoL Interpersonal Relationships. Similarly, DASS-21 Stress correlated positively with PANAS Positive Affect and negatively with WHOQoL Environment, suggesting that reductions in stress symptoms may translate into greater emotional engagement and improved perception of external conditions. These findings collectively reinforce the hypothesis that Melissa phospholipids supplementation modulates mood and stress through both neuroendocrine and psychosocial pathways. The significant intra-scale correlations for PANAS (positive and negative affect), WEMWBS, and WHOQoL-BREF further indicate that improvements in emotional tone and perceived quality of life were consistent and sustained across the intervention. In particular, WEMWBS correlated positively with WHOQoL Physical Health, suggesting that enhancements in psychological well-being were accompanied by parallel perceptions of improved physical functioning. The strong intra-domain associations observed for WHOQoL Physical Health and Mental Health highlight the internal coherence and reproducibility of these constructs over time.

These psychometric and endocrine findings point toward a synergistic relationship between reduced HPA axis activation and enhanced emotional and environmental adaptation. The observed associations suggest that the clinical improvements documented in the primary study, particularly regarding sleep quality and mood, are supported by measurable neuroendocrine modulation. In that subgroup, Melissa phospholipids supplementation was associated with a concurrent reduction in salivary cortisol and an improvement in sleep and mood. While significant cross-domain correlations were observed at an uncorrected level, the direct correlation between changes in Cortisol and Psychometric scores remained as a directional trend. Therefore, while the modulation of the HPA axis is a plausible mechanism underlying the clinical benefits, this link remains exploratory due to the limited sample size.

From a mechanistic perspective, *M. officinalis* is rich in polyphenolic compounds such as rosmarinic acid and caffeic acid, which have demonstrated GABAergic and antioxidant activity in preclinical studies (11, 12). The phospholipid formulation employed in this trial may potentiate central effects on stress reactivity and sleep regulation. Previous in vitro research on Melissa phospholipids confirmed neuroprotective, anxiolytic, and anti-inflammatory properties (22), while clinical evidence supports its role in improving sleep continuity and reducing insomnia severity (9, 10). The present findings extend these clinical results by demonstrating parallel endocrine and psychological improvements, offering biological plausibility for the anxiolytic and sleep-promoting observations recorded during supplementation of Melissa phospholipids. Furthermore, they suggest that salivary cortisol could serve as a feasible biomarker for monitoring the neuroendocrine response to dietary supplementation targeting stress-related disorders. Nonetheless, while initial analyses suggested widespread associations between cortisol dynamics and mental health scores, the application of FDR correction suggests caution. The observed psychophysiological coherence should be interpreted as exploratory, given the small sample size.

Nevertheless, several limitations should be acknowledged. This secondary analysis involved a small subgroup (*n* = 12), which restricts statistical power and generalizability. The lack of a placebo or lower-dose comparator precludes definitive attribution of the observed changes solely to *M. officinalis* supplementation. Although multiple time points were analyzed, cortisol was assessed only during daytime hours, limiting the ability to evaluate diurnal variation or cortisol awakening response (CAR), which are key markers of HPA axis regulation. Future studies should include more frequent sampling over consecutive days to capture the full circadian rhythm. It would be also interesting, by increasing the subjects’ number, to study the potential impact of age on the results obtained. A primary limitation is the absence of a parallel control group for the cortisol endpoint, which limit definitive causal attribution. The observed reductions may be influenced by regression to the mean or natural fluctuations in HPA axis activity over the 3-week period. Further randomized controlled studies are needed to deeply explore the concrete biological effects. Another limitation concerns the correlational nature of the findings. Although the results indicate significant associations between cortisol modulation and psychometric improvements, causality cannot be inferred. It remains unclear whether cortisol modulation precedes or follows improvements in sleep and mood, or whether both arise from a shared neurochemical mechanism. Furthermore, our sampling protocol (three timepoints) and the lack of formal slope modeling limit the claim of a full restoration of the diurnal rhythm. Future studies should employ more frequent sampling to calculate the CAR and diurnal slope accurately. Finally, the short intervention period (3 weeks) limits conclusions regarding the persistence of these effects. Even though, the magnitude of cortisol reduction observed after 3 weeks suggests that even relatively short-term supplementation may have measurable neuroendocrine effects, which could translate into meaningful clinical benefits. Long-term, randomized, placebo-controlled studies with larger sample sizes and integrated biomarker panels, including inflammatory cytokines, oxidative stress markers, and neurotrophic factors, are warranted to confirm the proposed pathways of action and to further strengthen the clinical evidence on the mechanisms through which Melissa phospholipids exert their effects.

## Conclusions

This secondary analysis of the principal study (14) demonstrates that 3 weeks of daily supplementation with a standardized phospholipid-based *M. officinalis* extract is associated with a progressive and clinically relevant reduction in daily salivary cortisol levels in adults with poor sleep quality and emotional distress. The modulation of HPA axis activity was paralleled by improvements in sleep quality and reductions in depression and stress scores, suggesting a potential mechanistic link between cortisol modulation and the clinical benefits observed in the primary study. These findings complete the previous clinical evidence and mechanism of action supporting the role of Melissa phospholipids in stress regulation and sleep–mood interactions. This research, even if exploratory, reinforces the concept that nutraceutical approaches can modulate key stress-response pathways and complements previous evidence of their effects on subjective outcomes. Larger randomized, placebo-controlled trials with extended follow-up and comprehensive diurnal cortisol profiling are warranted to confirm our results and clarify their implications for clinical practice.

## Data Availability

The data presented in this study are available on request from the corresponding author.
